# Survey and Analysis of Age and Antiplatelet/Anticoagulation Medication Status of the Desert Regional Medical Center Emergency Department Patients

**DOI:** 10.7759/cureus.15673

**Published:** 2021-06-15

**Authors:** Chao Li, Yasir R Khan, Javed Siddiqi

**Affiliations:** 1 Neurosurgery, College of Osteopathic Medicine, Des Moines University, Des Moines, USA; 2 Neurosurgery, Desert Regional Medical Center, Palm Springs, USA; 3 Neurosurgery, Riverside University Health System Medical Center, Moreno Valley, USA; 4 Neurosurgery, Arrowhead Regional Medical Center, Colton, USA; 5 Neurosurgery, California University of Science and Medicine, Colton, USA

**Keywords:** antiplatelet, anticoagulation, emergency department, age, aspirin

## Abstract

Objective

To delineate whether the geographical region served by the Desert Regional Medical Center (DRMC) emergency department (ED) in Palm Springs, California has a higher percentage of patients taking antiplatelet and/or anticoagulant medications during the winter season due to the seasonal influx of elderly patients.

Method

We conducted a prospective study in the patients seen in the DRMC ED between November 12, 2019 and November 14, 2019, and those patients were anonymously surveyed regarding their outpatient use of antiplatelet and anticoagulant medications. Verbal consent for participation was obtained. All the registered patients at the DRMC ED during the prescribed period of time were included. While the patients who were sent home by a triage nurse for non-medical issues or minor ailments requiring no treatment from ED providers and patients who refused to participate or were unable to consent were excluded. Age, gender, medication status including whether on any medication, antiplatelet and anticoagulant use, including dosage, and last dose time were recorded.

Results

Of the 110 consecutive patients arriving to the ED during the study period, 11 patients were unwilling or unable to provide consent for participation. Of the 99 participating patients, 37 (37%) were over 65 years old. The most common antiplatelet and/or anticoagulant medication taken by the patients was aspirin (24 patients). Aspirin was most commonly taken as once a day, in a total of 11 patients (11%). Thirty patients were taking one or more antiplatelet or anticoagulant medications.

Conclusion

ED at a hospital in Palm Springs, California has more elderly visits as compared to the national and California state averages. However, antiplatelet usage in elderly patients in our study was lower than the national average.

## Introduction

Palm Springs, California is a metropolitan city with a summertime population of 400,000. In the winter months, the population has been shown to swell up to 800,000 people, largely due to tourists making short-term visits and longer-term inhabitants known as seasonal “snowbirds” from northern states and countries [1]. Desert Regional Medical Center (DRMC) is a Joint Commission-certified primary stroke center and is the only Level II trauma center in the Coachella Valley, covering more than 8000 square miles. This dynamic demographic change makes DRMC, the area’s only comprehensive stroke center, one of the busiest stroke centers in California, particularly during the winter season. The emergency department (ED) is the main healthcare access point for DRMC’s stroke patients, most of whom arrive directly to the ED as a stroke code/alert case or as a transfer from an outside hospital.

For decades, antiplatelet and/or anticoagulation medications, such as aspirin, have been used for treatment and/or prevention of stroke, myocardial infarction, and other cardiac conditions [2]. Almost over 50% of elderly patients have been found taking aspirin daily or every other day with/without the recommendation of a physician. The most recent study shows that over 30 million patients in the USA take aspirin [2]. Notably, antiplatelet and anticoagulant medications increase the risk of cerebral hemorrhage [2]. This paper looks into whether the ED visits in a community hospital differ by age, as well as antiplatelet and anticoagulant use, from the national and state level.

## Materials and methods

This study was approved by the Institutional Review Board. Patients were given a hard copy with information about the study. All patients who came to the ED were asked to fill out the survey. Patients were excluded if they were sent home by a triage nurse for non-medical issues or minor ailments not requiring the attention of a physician such as prescription refill, headache without warning symptoms, minor injury due to sports, skin abrasion, minor fever, and so on, and patients who refused to participate or were unable to provide consent due to their medical or mental status.

Data collected included age, gender, general medication status, current antiplatelet and anticoagulant medication use, and antiplatelet and anticoagulant dose.

Descriptive statistics were run. Fisher's exact test, chi-square test, or analysis of variance, as applicable, was done to compare groups.

## Results

The study included 99 patients. Table 1 shows the patients' age and gender details.

**Table 1:** Subjects’ categorization by age and gender

Age	Number
<21	12
21-60	44
61-80	32
>80	11
Gender	
Male	62
Female	37

Table 2 shows the number of subjects who were on general medication, and antiplatelet and anticoagulant medications.

**Table 2:** Subjects’ medication status as well as dosage of antiplatelet and anticoagulant medications

Medications	Number
Currently not on any medication	38
Currently on medication but no antiplatelet or anticoagulants	29
Currently on medication with antiplatelet and/or anticoagulants	32
Aspirin 81 mg every day	11
Aspirin 81 mg + clopidogrel 75 mg every day	5
Rivaroxaban 20 mg every day	3
Aspirin 81 mg every other day	2
Aspirin 81 mg + rivaroxabon 20 mg	2
Aspirin 325 mg every day	1
Aspirin 325 mg + clopidogrel 75 mg every day	1
Clopidogrel 75 mg twice a day	1
Aspirin 81 mg + apixaban 5 mg every day	1
Apixaban 5 mg twice a day	1
Dabigatran 150 mg twice a day	1
Aspirin + dabigatran 150 mg twice a day and clopidogrel 75 mg twice a day	1
Enoxaparin 100 mg twice a day	1

Aspirin usage was analyzed separately. This included patients who were taking aspirin alone or with other anticoagulants (Table 3).

**Table 3:** Aspirin usage by age

Age	Number of subjects taking aspirin (%)
<18	0 (0)
18-60	7 (7.07)
61-80	14 (14.14)
>80	3 (3.3)
Total	24 (24.24)

There was a statistical significance found between the age groups (P = 0.006), though there was no significance found in the groups when compared to numbers from another study [2].

## Discussion

The study shows that the percentage (37%) of elderly patients visiting the DRMC ED is higher than the national average for ED visitors in this demographic. A 2012 study by the Centers for Disease Control and Prevention demonstrated that the national average percentage of ED visits made by elderly patients aged 65 years and older was 16%, with the percentage of ED visits made by elderly persons in California at that time being 22% [3].

Additionally, the number of patients visiting the ED in our survey who were less than 18 years old totaled 9 (9%), significantly lower than the national average of 21% and the California average of 19% [3]. Despite the higher percentage of elderly patients in our study, the usage of aspirin in this patient group was lower compared to the national average [4].

There was a statistical significance found between the age groups taking aspirin, either alone or in combination with other anticoagulants (P < 0.05). This significance was anticipated since aspirin is primarily used for prevention of stroke and cardiovascular diseases after 40-50 years of age [2,4,5].

There are several limitations of this study. The brevity of the time window during which the survey was performed, and the limited number of patients surveyed may have impacted our results. There are no published data regarding specific anticoagulation usage in general population in state or national level in the USA. The studies we found focus on either specific group such as skilled nursing home residents or comparison between various anticoagulation medication usage in the USA [6,7]. As such, we plan to start a multiple-phase study to follow our initial findings. In phase 2, we will survey another 100 patients over the age of 65 years consecutively arriving to the DRMC ED to assess their medication status, including antiplatelet and anticoagulant medications. In phase 3, 100 non-traumatic intraparenchymal hemorrhage patients consecutively arriving to the DRMC ED will be surveyed regarding their antiplatelet and anticoagulant medication use. The final goal of the study will be to investigate if the rate of antiplatelet and anticoagulant medication use among elderly patients in the California Coachella Valley exceeds that of the national average, and furthermore, if there is a correlation between higher usage of these medications among this population and non-traumatic intraparenchymal hemorrhage.

## Conclusions

DRMC ER patients have a higher percentage of elderly patients compared to the national and California average of patients visiting the ED . However, despite the higher percentage of elderly patients, the prevalence of aspirin usage in our study was lower than the national average.
